# Sterile endophthalmitis after intravitreal injection of triamcinolone acetonide: case report and literature review

**DOI:** 10.22336/rjo.2024.02

**Published:** 2024

**Authors:** David-Ionuț Beuran, Mioara-Laura Macovei, Cătălin Cornăcel, Ioana Ruxandra Boca

**Affiliations:** * Department of Ophthalmology, „Dr. Carol Davila” Central Military University Emergency Hospital, Bucharest, Romania; ** „Carol Davila” University of Medicine and Pharmacy, Bucharest, Romania; *** Clinical Emergency Eye Hospital, Bucharest, Romania

**Keywords:** sterile endophthalmitis, triamcinolone acetonide, macular edema, uveitis

## Abstract

**Objectives:** The first purpose is to present the diagnosis and therapeutic approach in a patient with sterile endophthalmitis associated with triamcinolone acetonide injection. The secondary objective is to assess the incidence of this complication and to summarize the risk factors described in the literature.

**Case presentation:** A 76-year-old male patient presented for painless, unilateral, decreased visual acuity, four days after cataract surgery and simultaneously intravitreal triamcinolone acetonide injection for diabetic macular edema in the right eye. The diagnosis of sterile endophthalmitis was made. Eight days after the presentation, the symptoms subsided, the maximum corrected visual acuity reaching that before the procedures.

**Discussions:** The incidence of sterile endophthalmitis varies in the literature between 0% and 23.8%. Visual prognosis is good, although the pathogenesis is not fully understood. Preservatives in injectable solutions have been suggested, however, there are studies in which inflammation was also present with preservative-free products. The particle size of triamcinolone was analyzed, demonstrating an association between smaller particles and an increased frequency of adverse reactions of this type. History of uveitis, posterior capsule rupture following cataract surgery, and Irvine-Gass syndrome are other associations described.

**Conclusion:** The physiopathological mechanism of sterile endophthalmitis is not fully understood. However, the visual prognosis is good, the final vision being dependent on the underlying pathology.

## Introduction

Triamcinolone acetonide is a white, crystalline, water-insoluble corticosteroid. It has an anti-inflammatory effect 5 times stronger than hydrocortisone. Due to its anti-edematous and anti-angiogenic effect, it has been widely used off-label in the case of pathologies involving neovascularization or alteration of the blood-ocular barrier [**1**].

From the situations in which it has been used, we specify diabetic macular edema, age-related macular degeneration, macular edema secondary to uveitis, Irvine-Gass syndrome, macular edema secondary to occlusion of the retinal vein branch or the central vein of the retina [**2**-**4**].

Intravitreal injection provides an increased concentration of corticosteroids in the vitreous with minimal systemic adverse effects [**5**].

The main side effects include secondary intraocular hypertension, secondary cataract, and endophthalmitis [**6**].

Endophthalmitis can be infectious, secondary to a pathogen, pseudo-endophthalmitis, due to the precipitation of triamcinolone crystals, or sterile endophthalmitis represented by an inflammatory reaction in the absence of a pathogen [**5**,**7**].

## Case presentation

*Patient data*

A 76-year-old Caucasian male presented with sudden, painless, decreased visual acuity in the right eye four days after cataract surgery and concomitant intravitreal injection of triamcinolone acetonide.

*History*

**Fig. 1:**
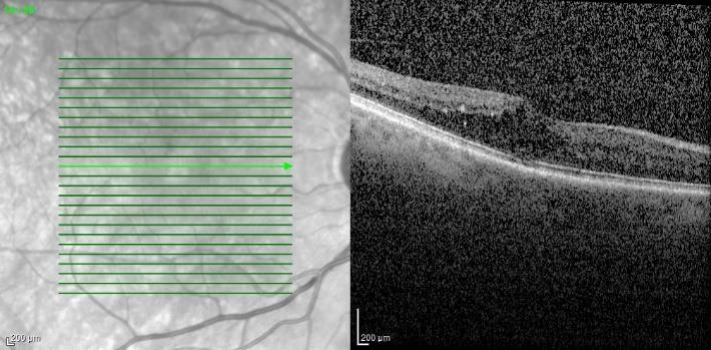
Right eye: optical coherence tomography

Three months before, the patient complained of decreased visual acuity in the right eye. From the personal history, type II diabetes should be mentioned. Following the ophthalmological examination, the diagnoses of diabetic macular edema and posterior subcapsular cataract were concluded. It was decided to start intravitreal injections with anti-vascular endothelial growth factor agents (aflibercept). Two injections were given at an interval of one month. The optical coherence tomography appearance of diabetic macular edema remained unchanged (**Fig. 1**) and best-corrected visual acuity decreased from 0.3 to 0.2 secondary to cataract progression. Cataract surgery with the implantation of a monofocal intraocular lens and concomitant intravitreal injection of 0,1 ml triamcinolone acetonide 40 mg/ml was performed. The left eye showed no pathological changes and maintained a best-corrected visual acuity of 1 throughout the follow-up.

*Examination results*

The patient had a visual acuity of 0.15 in the right eye, and an intraocular pressure of 12 mmHg, and the refraction could not be measured.

**Fig. 2:**
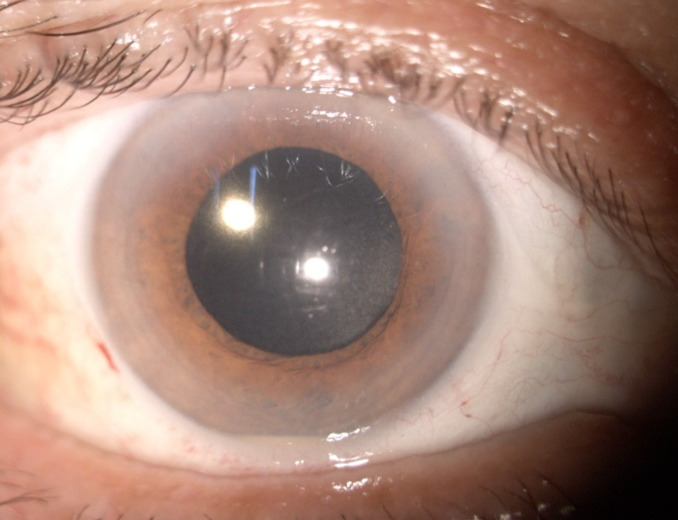
Right eye: first day of examination

**Fig. 3:**
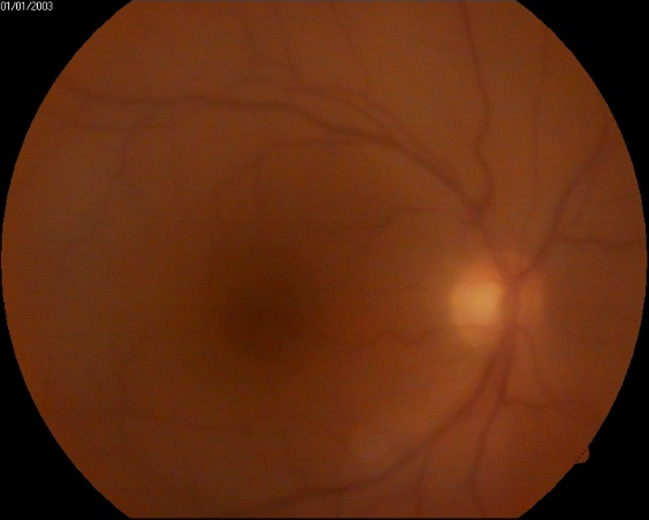
Right eye: first day of examination

Examination of the anterior pole revealed Descemet’s membrane folds, fine hypopyon, flare +1 in the anterior chamber, well-positioned intraocular lens, and diffuse punctate opacities in the anterior vitreous (**Fig. 2**). The fundus was visualized with difficulty and grade 2 mild vitreous haze was observed (**Fig. 3**).

*Initial diagnosis*

After the initial examination, the diagnoses were made for the right eye: sterile endophthalmitis, posterior chamber intraocular lens, diabetic macular edema, and for the left eye: hyperopia, and presbyopia.

*Paraclinical investigations*

**Fig. 4:**
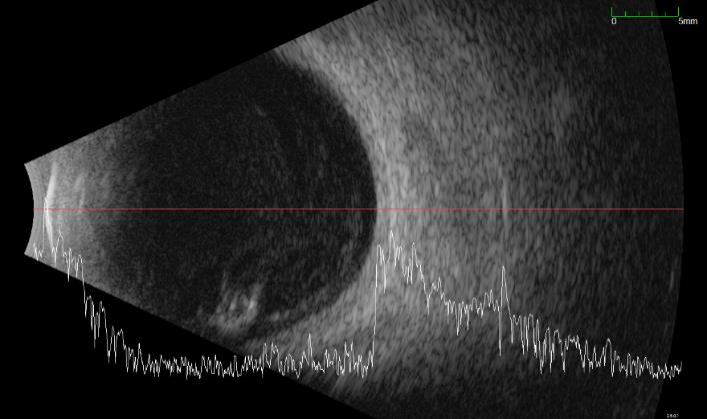
Right eye: ocular-orbital ultrasound, nasal transverse section, first day of examination

The oculo-orbital ultrasound detected numerous small, unsystematized echoes of subretinal intensity, homogeneously arranged throughout the vitreous cavity. Inferiorly, in the temporal and nasal transverse sections, an area with an echo of increased intensity, compared to the previously described structures, but subretinal, organized, was observed, which represented triamcinolone located inferiorly (**Fig. 4**).

**Fig. 5:**
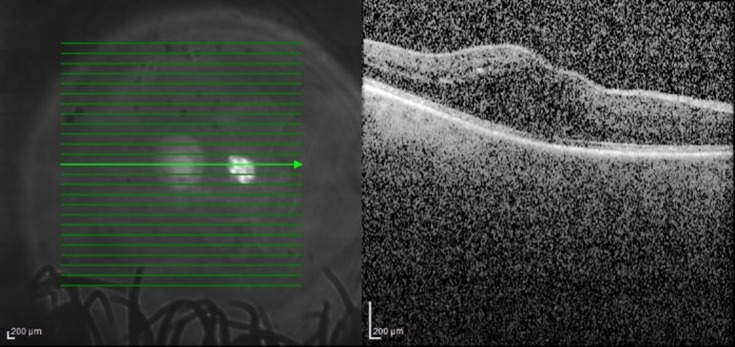
Right eye: optical coherence tomography, first day of examination

The optical coherence tomography examination of the macula was performed with difficulty. An increase in the amount of intraretinal fluid was observed (**Fig. 5**). 

Paraclinical investigations supported the initial diagnoses. Thus, the initial diagnosis became the positive diagnosis.

*Differential diagnosis*

It is done with anterior segment toxicity syndrome. As pro arguments, we had Descemet’s membrane folds, hypopyon, flare 1+, and cataract operated four days before. The cons were the following: the syndrome usually appears in 6-24 hours, the pain is present and is moderate-severe, the corneal edema is diffuse and more pronounced, severe reaction in the anterior chamber, and the vitreous is not involved.

Infectious endophthalmitis was the second pathology that was discussed for the differential diagnosis. The pros we had were the hypopyon, flare 1+, vitreous haze grade 2, operated cataract, and intravitreal triamcinolone acetonide injection four days before. The cons were: the pain is usually present and is moderate-severe, vision is lower (perceives light or perceives the movement of the hand), severe reaction in the anterior chamber, severe inflammation in the vitreous, and different ultrasound appearance.

*Treatment*

The patient was already on treatment with netilmicin 0.3% and dexamethasone 0.1% ophthalmic solution and artificial tears with trehalose 3% and sodium hyaluronate 0.15%, 5 times a day. It was decided to prepare and initiate treatment with fortified antibiotics: fortified ceftazidime 50 mg/ml, fortified gentamicin 15 mg/ml, and fortified vancomycin 50 mg/ml. These were administered 1 drop per hour and stopped after 48 hours.

*Evolution*

The next day, the patient presented with a vision of 0.1. Hypopyon did not increase in height, flare 1+ in the anterior chamber, and vitreous haze remained at grade 2. Third-day vision slightly improved, 0.15, hypopyon gone, flare 1+, vitreous haze grade 2. On the 8th day after the presentation, respectively the 12th after the surgery and injection, the vision was 0.3, the intraocular pressure remained at the value of 12 mmHg, and the refraction could be measured (spherical equivalent 0).

**Fig. 6:**
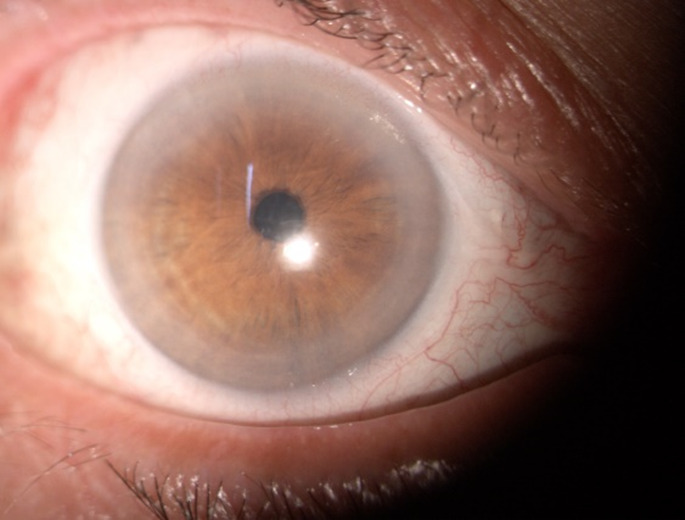
Right eye: 8th day of examination

**Fig. 7:**
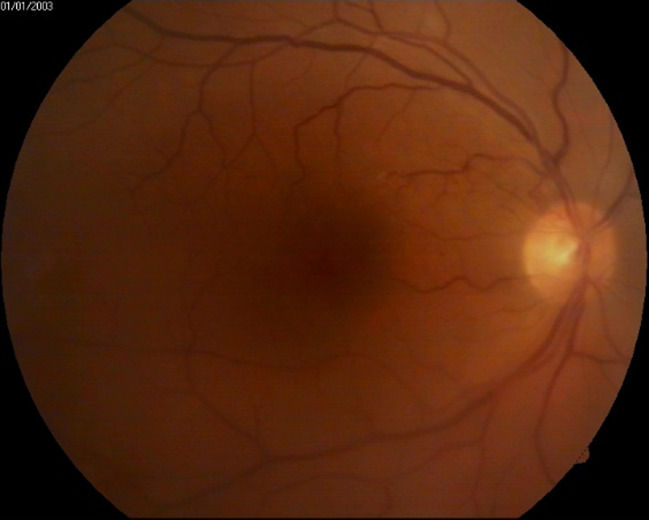
Right eye: 8th day of examination

The flare and vitreous haze were gone (**Fig. 6**,**7**). Diabetic macular edema remained at the same level as on the first day of examination.

## Discussions

*Incidence*

IThere are studies in the literature describing the administration of intravitreal triamcinolone acetonide for various pathologies of the posterior pole. The incidence of sterile endophthalmitis ranged from 0% to 23.8% in 14 studies [**2**-**4**,**8**-**18**] (**Table 1**).

**Table 1:** The incidence of sterile endophthalmitis in 14 studies

Indication	Injected eyes	Sterile endophthalmitis	Percent
Diabetic macular edema	43	1	0,02% [**2**]
Macular edema of various etiology	310	6	1,9% [**3**]
Macular edema of various etiology	21	5	23,8% [**4**]
Macular edema of various etiology	18	3	7% [**8**]
Macular edema of various etiology	81	0	0% [**9**]
Diabetic macular edema	69	0	0% [**10**]
Macular edema of various etiology	111	1	0,9% [**11**]
Macular edema of various etiology	929	6	0,6% [**12**]
Macular edema of various etiology	102	0	0% [**13**]
Diabetic macular edema	30	1	3,3% [**14**]
Macular edema of various etiology	64	0	0% [**15**]
Diabetic macular edema	210	6	2,8% [**16**]
Macular edema of various etiology	922	8	0,8% [**17**]
Macular edema of various etiology	554	11	1,9% [**18**]

*Clinical aspects*

Moshfeghi et al. described a study with a total of 922 intravitreal injections of triamcinolone acetonide and 8 cases of sterile endophthalmitis. Their median presentation time was 1.5 days (with a range of 1 to 7 days). Median visual acuity at presentation was 20/563 (range between 20/80 and perceived light). The median follow-up time after the event was 5.9 months (range 4 to 9 months) with a median visual acuity at the last assessment of 20/75 (range 20/40 on finger counts) [**17**].

Sterile endophthalmitis presents milder symptoms compared to infectious endophthalmitis. The pain is usually absent or mild and the decrease in visual acuity is not as severe. The time of presentation to the hospital is faster than in the case of infectious endophthalmitis, 1-3 days [**5**]. 

Sterile endophthalmitis associated with triamcinolone acetonide injection has no specific treatment. In general, it consists of corticosteroids, antibiotics, and cycloplegics [**5**,**19**,**20**]. The visual prognosis is good with vitritis resolution on average after two weeks and final vision is dependent on the underlying disease [**1**,**17**,**19**].

*Risk factors*

In a study of 310 eyes undergoing intravitreal injection of triamcinolone acetonide for various pathologies: age-related macular degeneration, diabetic retinopathy, macular edema secondary to uveitis, Taban et al. described 6 cases (1.9%) of sterile endophthalmitis. Of the 6 cases, 4 had a history of uveitis, and of the 310, 20 had such antecedents. All 6 patients presented within 3 days of injection. The median best-corrected acuity before injection was 20/100, and the final acuity was 20/80. The study found that patients with a history of uveitis may have an increased risk of sterile endophthalmitis after intravitreal injection of triamcinolone [**3**].

Wang et al. described in their study 21 injected eyes, of which 5 had sterile endophthalmitis (23.8%). Of the 5, 3 had pseudophakia with damage to the posterior capsule, and 4 had the diagnosis of Irvine-Gass syndrome. The rate of sterile endophthalmitis was significantly higher in patients with artificial lenses who had the posterior capsule affected (p=0.0075) and in patients with Irvine-Gass syndrome (p=0.0008). Final visual acuity did not decrease after the episode of sterile endophthalmitis [**4**].

Jonisch et al. described an increase in the incidence of sterile endophthalmitis after triamcinolone acetonide injection between May 1 and July 31, 2006. Between January 1, 2005, and July 31, 2006, 554 eyes were injected, and 11 (1.9%) sterile endophthalmitis were reported. Of the 11, 9 occurred between May 1 and July 31, 2006, when 97 eyes were injected. The incriminated batch was analyzed, but no bacterial endotoxins were detected [**18**].

Șuta et al described a series of 3 cases with the diagnosis of intermediate uveitis, who were given intravitreal injections with triamcinolone acetonide. All patients presented 24h post-injection with low best-corrected vision, ranging between perceived hand movement and 0.1. Treatment was with topical antibiotics, prednisolone acetate, and cycloplegic drops. The vitritis resolved in 3 weeks in one case and in 4 in the other 2 [**19**].

The pathogenesis is not fully understood. One of the hypotheses is the occurrence of an inflammatory reaction to the preservatives [**5**]. However, some studies described the adverse reaction also in the case of the administration of triamcinolone acetonide without preservatives [**21**]. Triamcinolone particle size was evaluated in another study, which demonstrated that smaller particles were associated with an increased rate of sterile endophthalmitis [**21**]. One study incriminated the presence of bacterial endotoxins, but these were ultimately absent [**18**]. Another study demonstrated associations with a history of uveitis [**3**], and another with posterior capsule damage following cataract surgery and Irvine-Guss syndrome [**4**]. Another argument for the association with uveitis is a series of 3 cases of sterile endophthalmitis, the patients having intermediate uveitis as the underlying disease [**19**].

## Conclusion

Although risk factors for the development of sterile endophthalmitis after triamcinolone acetonide injection have been described, the mechanism of occurrence is not fully understood. The visual prognosis is favorable, and the final vision depends on the underlying pathology.

**Conflict of Interest Statement**

The authors state no conflict of interest. 

**Informed Consent and Human and Animal Rights Statement**

Informed consent has been obtained from the patient included in the case report. 

**Authorization for the use of human subjects**

Not applicable. 

**Acknowledgments**

None. 

**Sources of Funding**

None.

**Disclosures**

None. 
